# High Heritability Is Compatible with the Broad Distribution of Set Point Viral Load in HIV Carriers

**DOI:** 10.1371/journal.ppat.1004634

**Published:** 2015-02-06

**Authors:** Sebastian Bonhoeffer, Christophe Fraser, Gabriel E. Leventhal

**Affiliations:** 1 Institute of Integrative Biology, ETH Zurich, Zurich, Switzerland; 2 Department of Infectious Disease Epidemiology, Imperial College London, London, United Kingdom; University of North Carolina at Chapel Hill, UNITED STATES

## Abstract

Set point viral load in HIV patients ranges over several orders of magnitude and is a key determinant of disease progression in HIV. A number of recent studies have reported high heritability of set point viral load implying that viral genetic factors contribute substantially to the overall variation in viral load. The high heritability is surprising given the diversity of host factors associated with controlling viral infection. Here we develop an analytical model that describes the temporal changes of the distribution of set point viral load as a function of heritability. This model shows that high heritability is the most parsimonious explanation for the observed variance of set point viral load. Our results thus not only reinforce the credibility of previous estimates of heritability but also shed new light onto mechanisms of viral pathogenesis.

## Author Summary

Following an initial peak in viremia, the viral load in HIV infected patients settles down to a set point which remains more or less stable during chronic HIV infection. This set point viral load is one of the key factors determining the rate of disease progression. The extent to which it is determined by the virus versus host genetics is thus central to developing a better understanding of disease progression. Here we develop an analytical model that describes the changes of the distribution of set point viral load in the HIV carrier population over a full cycle of transmission. Applying this model to patient data we find that the most parsimonious explanation for the observed large variation of set point viral load across HIV patients is that set point viral load is highly heritable from donors to recipients. This implies that set point viral load is to a considerable extent under the genetic control of the virus.

## Introduction

The time course of viral load in HIV infected patients follows a characteristic pattern. During primary infection the viral load rapidly grows to very high levels. The peak viremia is attained within the first few weeks of infection. Thereafter the viral load declines rapidly over a period of several months and eventually settles down at a much lower level referred to as the viral set point. Set point viral load (spVL) is a central characteristic of the course of the disease. Firstly, the virus load measurements do fluctuate in patients, the time average of the viral load remains remarkably close to the spVL in most of patients over the time scale of several years [1, 2]. Secondly, higher spVL is associated with faster disease progression [3].

The stability of spVL within patients is in strong contrast to the enormous variation in spVL observed between patients. While variation in spVL between patients ranges over 3–4 orders of magnitude [3–6], the time trend over longitudinal viral load measurements typically changes by less then 0.1 log per year [1, 2]. Given that spVL is a key predictor of disease progression, there is considerable interest in identifying the host and viral genetic factors underlying the variation in spVL.

A well known example for the influence of naturally occurring variation in human genetic factors on viral load is the Δ32 deletion in the *CCR5* gene [7]. Moreover polymorphisms in HLA-B and C alleles have been associated with variance in virus load and genome-wide association studies (GWAS) showed that about 20% of the variance in log spVL can be attributed to specific single nucleotide polymorphisms [8–11]. 20% is likely a lower bound for the overall contribution of host genetic factors, because GWAS generally suffer from the problem that they can only identify common genetic variants with strong effects and do not account for epistatic effects between host genes [12].

Natural variation in the virus can also affect spVL. For example the transmission of a *nef*-deficient virus through a contaminated blood sample resulted in a low viral load in the recipients [13]. Moreover, several studies have reported a correlation between predicted replicative capacity and viral load [14–16]. As this prediction is based only on the viral genotype a patient carries, this implies that naturally occurring variation in viruses does affect viral load. A number of recent studies attempted to estimate the contribution of the viral genotype to the variation in spVL by quantifying the statistical association of viral load between donors and recipients either directly in donor-recipient pairs or through phylogenetic analysis [17–22]; for reviews see Müller et al. [23] and Fraser et al. [24]. A meta-analysis of previously published donor-recipient studies correcting for various co-factors such as age and sex yielded a heritability of 33% with a 95% confidence interval of 20–46% [24]. The two studies that inferred heritability based on phylogenetic methods provided the most extreme estimates with 5.7% reported by Hodcroft et al. [22] and 59% reported by Alizon et al. [21]. While the phylogenetic approaches have an advantage over the donor-recipient based approaches in that they can use much larger patient populations, it is currently unclear to what extent the underlying assumptions of the phylogenetic approaches of no selection and high frequency of sampling affect the robustness of these results.

The discrepant estimates call for a better quantitative understanding of the underlying factors determining heritability of log spVL in HIV. To this end we develop here a quantitative model that describes the change of the distribution of log spVL in a patient population in relation to heritability over a full transmission cycle. The model extends the approach of Shirreff et al. [25] and is similar in spirit to the integral projection models in ecology that are used to describe the temporal changes of distributions of a continuous phenotypic trait in populations [26–28]. In contrast to many applications in ecology, the application to distributions of log spVL has the advantage that all relevant processes and populations for which data are available, are numerically well approximated by a Gaussian function. This fact enables us to obtain complete analytical understanding of how spVL changes through time based on a model parametrized by available data.

## Results

**Fig 1:**
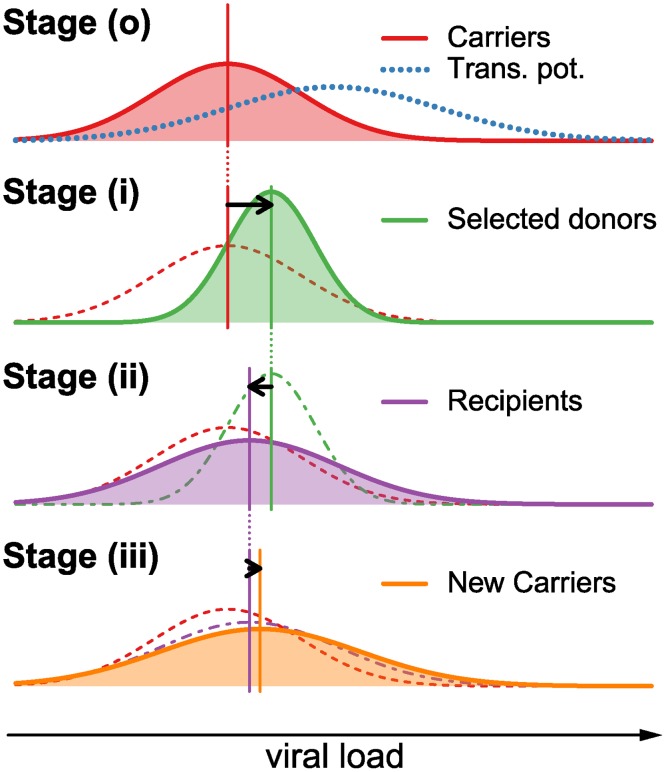
Graphical example of the change in the distribution of log spVL in the population over one reproduction cycle. During one full reproduction cycle, the distribution goes through the following steps: (o) The log spVL distribution within a population follow a Gaussian function with mean *M*_*C*_ and variance *V*_*C*_ (red curve). The transmission potential (blue dotted line) selects a subset of this population as donors (see Equation 5). (i) The transmission potential selects donors from the carrier population in (o) with mean *M*_*D*_ that lies between the mean of the carriers, *M*_*C*_, and the mean of the transmission potential, *μ*_*o*_. The resulting variance in log spVL in the selected donors is smaller than in the carrier population (see Equation 7). (ii) The selected donors transmit to new hosts, thus randomizing the host/environmental contributions and lowering the population mean log spVL and increasing the population variance. The variance is further increased by a transmission bottleneck and sampling effect on the level of the individual donors.(iii) Within-host evolution of log spVL may further increase or decrease the population mean, while always increasing or not affecting the variance. This completes a full reproduction cycle. In equilibrium, the individual changes in mean and variance in stages (i), (ii) and (iii) is such that the overall change in mean and variance from stage (o) to (iii) is zero.

We consider the change of the spVL distribution over one full reproduction cycle on the epidemiological level, i.e. from the current to the next generation of patients. We divide the patient population into “carriers” (HIV infected individuals prior to selection for transmission), “donors” (individuals that have been selected for transmission) and “recipients” (individuals that have just been infected by donors). Furthermore, we divide the reproduction cycle into three steps: (i) selection of donors from the carriers with replacement according to their transmission potential, (ii) transmission from donors to recipients, and (iii) intrahost evolution of the virus from the start of infection to the next transmission. Finally, we explicitly distinguish between factors contributing to set point viral load with regard to being transmissible (i.e. viral genetic factors) versus being non-transmissible (i.e. host genetic factors, environmental factors, or any interaction between host, virus, and the environment). A schematic overview over the effects of these steps on the distribution of log spVL is shown in Fig. 1.

In the Supplementary Materials we show how the change of the spVL distribution can be computed for any distribution over a full transmission cycle. If all populations and processes are well approximated by Gaussian functions, then an approximation to the resulting log spVL distributions can be computed analytically (see Methods and Supplementary Materials). Assuming that the population is in equilibrium we obtain for the mean, *M̃*_*C*_, variance, *Ṽ*_*C*_, and heritability, *h* ^2^, the following expressions:

$${\tilde{M}}_{C}\;=\;\mu_{o}+\mu_{i}\left(1+\frac{\nu_{e}+\nu_{o}}{{\tilde{V}}_{C}-\nu_{e}}\right),$$ (1)

$${\tilde{V}}_{C}\;=\;\frac{\nu_{t}+\nu_{i}}{2}\left(1+\sqrt{1+4\frac{\nu_{e}+\nu_{o}}{\nu_{t}+\nu_{i}}}\right)+\nu_{e},$$ (2)

$$h^{2}\;=\;1-\frac{\nu_{e}}{{\tilde{V}}_{C}}.$$ (3)

Here the parameters *μ*_*o*_ and *ν*_*o*_ characterize the transmission potential [6], i.e. the overall probability of a patient to transmit the infection as a function of log spVL (Fig. 1(o)–(i)). This transmission potential is given by the product of the rate of transmission per contact and the disease duration. As the former increases and the latter decrease with increasing spVL, the transmission potential has a maximum at intermediate levels of spVL [6]. The parameter *ν*_*e*_ gives the variance of the contribution of host/environmental effects on log spVL. The parameter *ν*_*t*_ describes the variance due to the bottleneck at transmission from donor to recipient, as a founder strain is selected randomly from the diverse population in the donor (Fig. 1(i)–(ii)). The parameters *μ*_*i*_ and *ν*_*i*_ describe the mean and variance of the contribution of intrahost evolution to log spVL (Fig. 1(ii)–(iii)).

Our model assumes that the bottleneck at transmission is neutral with regard to selection on set point viral load. Note, that the assumption is without loss of generality. This is important because there is evidence for selection at transmission [29], although it is unclear whether selection acts on spVL. Any selective effect at transmission, however, can be subsumed into the parameter *μ*_*i*_. Hence, the effect of selection is effectively incorporated in our model.

The parameter for the mean contribution by the host/environment, *μ*_*e*_, does not appear in equations 1 or 2. This is because the equations refer to the phenotypic value of spVL, i.e. the sum of the genetic contributions of the virus and the contributions from the host/environment. Any large environmental/host effect on the mean can always be compensated by correspondingly strong genetic effect of the virus on the mean but with opposite sign.

The above results are applicable if, (i) if the population is approximately in equilibrium, and (ii) all populations and processes are numerically well approximated by Gaussian functions.

**Fig 2:**
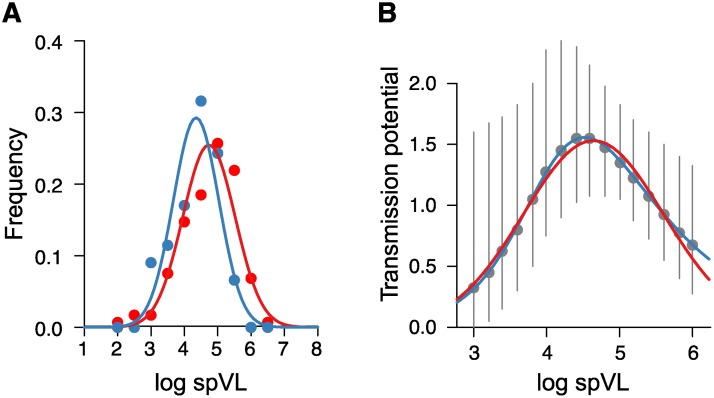
Viral load distributions and transmission potential estimated from patient cohorts as extracted from the corresponding graphs in Fraser et al. [6]. (**A**) The viral load distributions in the a Zambian and a Dutch cohort (see Fraser et al. [6]). The lines correspond to the best fits of a Gaussian to the distribution of log spVL. The null hypothesis that log spVL is normally distributed cannot be rejected based on a test that tests whether the residuals between model and fit themselves are normally distributed. The estimated mean and standard deviation are 4.74 and 0.61 for the Zambian data (red) and 4.35 and 0.47 for the Amsterdam data (blue). (**B**) The transmission probability according to the functions for transmissibility and duration of disease as a function of viral load as provided in Fraser et al. [6]. The grey circles and lines represent the mean and 95% confidence interval of the transmission potential as estimated from the Zambian and Amsterdam cohorts by Fraser et al. [6]. The blue line represents the corresponding theoretically derived transmission potential as provided by Fraser et al. [6]. The red line corresponds to the best fit of a log Gaussian to the estimated transmission potential. The parameters of the fitted log Gaussian are *μ*_*o*_ = 4.64±0.021 and *ν*_*o*_ = 0.96±0.025 (estimate ± standard deviation).

Assumption (i) has been discussed in detail previously [6, 25, 30, 31]. In essence, this assumption is supported by three observations. Firstly, the mean of the spVL distribution coincides with the optimum of the transmission potential (see Fig. 2 and Fraser et al. [6]). Secondly, the rate of change of spVL has decreased over the last 25 years [31]. Thirdly, the rate of evolution is sufficiently rapid such that a spVL that is optimal for transmission could have evolved over the course of the epidemic [25]. These findings suggest that the distribution of set point viral load is indeed approximately in equilibrium, which in turn makes it is plausible to assume that the environmental and genetic factors determining set point viral load are also in equilibrium.

Regarding assumption (ii), we note that a Gaussian function describes a distribution or process by a main effect (mean) and some variational noise (variance). Thus in absence of any better knowledge, a Gaussian distribution is a natural starting point to describe any process and simply represents a second order approximation to an unknown distribution. We can assess the validity of describing the distributions of spVL in carriers and the transmission potential graphically using available data. Inspection of Fig. 2A and Figure S1 in Supplementary S1 Text shows that the viral load amongst carriers is indeed numerically well approximated by a Gaussian with mean log spVL, *M*_*C*_ ≈ 4.5, and variance in log spVL, *V*_*C*_ ≈ 0.5. Also the fit of a Gaussian to the transmission potential (see Fig. 2B) is a very good approximation (mean *μ*_*o*_ ≈ 4.6 and variance *ν*_*o*_ ≈ 1.0), even though the transmission potential as estimated by Fraser et al. [6] is slightly right-skewed.

There are no data to inform the shape of the processes of transmission and intrahost evolution. Using a description that has a mean effect with some variation around this mean is natural. Nonetheless, we test the effect of numerical deviations from a Gaussian with the following simulations. Firstly, we use the exact right-skewed transmission potential as given by Fraser et al. [6]. The analytical approximations for the distribution of the population in equilibrium remain excellent when the substantial deviations of the transmission potential from a Gaussian are incorporated (see Figure S2). Secondly, we study the robustness towards deviations from Gaussian functions in the processes describing intrahost evolution and the transmission bottleneck. Even when both processes are strongly skewed, the analytical approximations for mean and variance are excellent (typically less than 2% deviation, see Figure S3 in Supplementary S1 Text).

To assess what heritability values are compatible with the observed mean and variance of log SPVL in the carrier population we take a simple approach that is in essence Approximate Bayesian Computing with rejection sampling. To this end we define plausible prior distributions for the parameters of the model. Sampling randomly from the priors we determine the resulting means and variances of log spVL in carriers and reject sets of parameters that lead to means and variances outside a defined permissible range. The set of accepted parameters gives the posterior distribution.

For the range of permissible mean log spVL we assume 4 < *M̃*_*C*_ < 5, which is compatible but somewhat larger than the observed range in the studies reported by Fraser et al. [6] and Geskus et al. [5] (see Fig. 2A and Supplementary Materials, Section E). For the permissible range of variances of log spVL we assume that 0.3 < *Ṽ*_*C*_ < 0.8, which again is compatible but somewhat larger than the values reported by Fraser et al. [6] and Geskus et al. [5] (see Supplementary Materials, Section E).

We use uniform priors for all parameters. The parameters *μ*_*o*_ and *ν*_*o*_, which describe mean and variance of the transmission potential, have thus far only been estimated only by a single peer reviewed study (Fraser et al. [6] and Fig. 2B; see also [32]). To account for uncertainty in the estimates of these parameters we use 4 < *μ*_*o*_ < 5 and 0.5 < *ν*_*o*_ < 1.5. Estimates for remaining parameters cannot be easily derived from the existing literature. To account for uncertainty in these parameters we assume 0 < *ν*_*e*_ < 1; −1 < *μ*_*i*_ < 1; 0 < *ν*_*i*_ < 0.3 and 0 < *ν*_*t*_ < 0.3.

**Fig 3:**
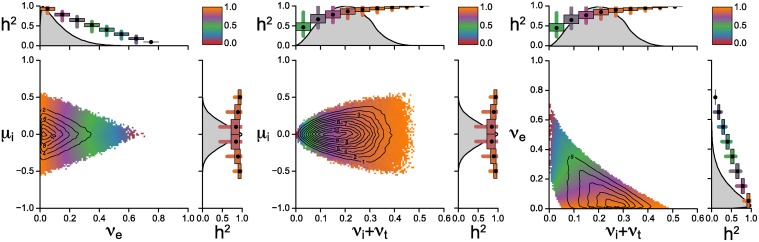
Posterior distribution of parameters from the rejection sampler. We report pairwise scatterplots of the parameters *μ*_*i*_, *ν*_*e*_ and the compound parameter *ν*_*i*_ + *ν*_*t*_, since these two parameters only appear as a sum in all equations for the mean, variance and heritability. 10^7^ random sets of parameter values are sampled randomly from the uniform priors described by the ranges on the *x* and *y* axes. Around 1.9% of the randomly generated parameter combinations yield values for mean and variance of log spVL that are compatible with the acceptance criterion 4 < *M̃*_*C*_ < 5, 0.3 < *Ṽ*_*C*_ < 0.8. The contour lines show the two-dimensional kernel density estimate of the posterior sample. The colours reflect the mean heritability of binned parameter combinations and are stacked such that points with lower heritability lie on top of points with higher heritability. The small plots to the top and right of the scatterplots show the posterior density estimate along a single parameter dimension, as well as the mean (black dot), and 50% (boxes) and 95% quantiles (lines) of heritabilities along those parameter dimensions. Most of the probability mass occurs at low values of *μ*_*i*_ and *ν*_*e*_.

Fig. 3 shows the posterior parameter distribution from the rejection sampling. Different colors in the scatter plots indicate different levels of mean heritability at given parameter combinations. The contour lines show the density of posterior distribution. The key result shown in the figure is that the majority of accepted parameter values result in high values of heritability (purple to orange color at contour lines of highest posterior densities). While low values of heritability are also compatible with the observed mean and variance of log spVL, they occur rarely in the posterior sample and are at the edges of the prior distributions (red to blue areas). The center of mass of the posterior sample is in areas with high heritability, higher in fact then what would seem compatible with current estimates of heritability and host genetic factors. There are two factors not included in this analysis: measurement error of spVL and prior knowledge of the host contribution to spVL. Increasing the measurement accuracy of spVL would increase heritability estimates based on both donor-recipient pairs and phylogenetic inference. Incorporating prior knowledge of the host genetic contribution would set an upper bound on the estimates of heritability in our analysis. Thus accounting for these two factors bring the center of mass of the heritability distribution closer to the measured values of heritability.

The figure also highlights that generally wider priors would not change the posterior distribution because parameter values at the upper end of priors are never accepted. The change of the mean capacity of the virus to induce spVL through intrahost evolution, *μ*_*i*_, is restricted to values smaller than 0.6 and decreases with increasing variance generated by the host/environment effects, *ν*_*e*_. Increasing *ν*_*e*_ corresponds to decreasing heritability (see eq. 3) and thus high levels of *μ*_*i*_ require high levels of heritability. The center of mass of the posterior sample suggests that the most parsimonious explanation of the observed mean and variance of log spVL implies both small intrahost evolution and high heritability.

One criticism leveled against the transmission potential as quantified in [6] is that it does not appropriately reflect transmissions occuring during the acute or the AIDS phase. In the Supplementary Material, Section F.3, we show that our quantitative results are robust towards using a corrected transmission potential.

## Discussion

The above analysis shows that the most parsimonious explanation of the observed distribution of spVL in HIV carrier populations requires high heritability of spVL. Although low heritability values are also compatible with the observed distribution of spVL in HIV carrier populations, parameter combinations resulting in these low values have a small probability and occur at the edge of the realistic parameter range. The skepticism with which the estimated heritability values have been met in the field suggests that the general expectation is that heritability of spVL should be low. In contrast, our analysis shows that high heritability values are not only compatible with, but are also the more parsimonious explanation of the observed distribution in spVL in HIV carrier populations.

Low heritability only occurs if the processes of intrahost evolution and the transmission bottleneck have a weak effect on spVL, i.e. if the parameters *μ*_*i*_, *ν*_*i*_ and *ν*_*t*_ are small. An intuition can be obtained by noting that in equilibrium the variance generating and variance eliminating processes balance out. The transmission potential only exerts weak selection on log spVL and therefore only marginally reduces variance. The decrease of variance by selection for transmission has to be compensated by an increase in variance by intrahost evolution and the transmission bottleneck. For too low heritability, the genetic variance generated by intrahost evolution and transmission bottlenecks would overwhelm the reduction of variance due to selection by the transmission potential. While there are to our knowledge no data that allow to estimate the variance generated at transmission, *ν*_*t*_, the posterior distributions of *μ*_*i*_ and *ν*_*i*_ are broadly compatible with the observed changes of virus load within patients [1, 2, 33].

Taken together our analysis suggests that the most parsimonious explanation of the distribution of log spVL is high *h* ^2^ but low *ν*_*t*_, *μ*_*i*_ and *ν*_*i*_. Hence, heritability is high while the processes of intrahost evolution and transmission bottleneck have a small effect on the capacity of the virus to modulate log spVL. High heritability implies a substantial genetic control of the log spVL by the virus. The observation that at the same time the contribution of intrahost evolution to spVL is small raises an interesting question: How can a strongly heritable trait show little intrahost evolution? Given the otherwise ample evidence for rapid intrahost evolution of HIV such as escape from drugs or the immune response, the absences of intrahost evolution of spVL is surprising. Generally a trait is expected to respond to selection, if (i) the trait is heritable, (ii) there is phenotypic variation of the trait in a population, and (iii) the trait is linked to fitness. That spVL is heritable has been reported previously [23, 24] and our analysis reinforces the credibility of these findings. That there is phenotypic variation in the control of spVL by the virus is plausible given the large genetic variation of the virus population within an individual. What remains is whether it is conceivable that the capacity of a viral genotype to induce spVL is only weakly linked to fitness. One hypothesis that could reconcile high heritability with little intrahost evolution is that variation in viral load between patients is in part due to virus-induced activation of target cells. Difference in activation rate of target cells has previously been argued to account for a substantial part of the variation in viral load [4]. Furthermore, if target cell activation is at least partially under the control of the virus, then this control may indeed be weakly linked to intrahost fitness. If the target cell activation is systemic (i.e. not locally confined to the inducing virus) then increased target cell activation increases the pool of susceptible cells, but the benefit of increased target cell activation is not confined to the producer virus. As a result selection for virus induced activation rate is expected to be neutral or nearly neutral [34]. Indeed, an explicit model of the evolution of log spVL for a virus induced control of target cell activation can reconcile high heritability with absence of intrahost evolution [35].

Our modeling approach is based on describing how the distribution of a continuous phenotypic trait, here log spVL, changes in a population over a full cycle of reproduction. This approach is closely related to the method of integral projection models, which has been developed and widely applied in ecology and population biology [26–28, 36, 37]. The approach can in principle describe how arbitrary distributions change over time as a function of processes such as selection and reproduction. Here we are able to obtain a full analytical description of the temporal change of the spVL distribution, because all relevant distributions and processes can be well approximated by Gaussian functions. We also show that our analytical results remain robust even for substantial deviations numerical deviations from Gaussian functions (see Supplementary Materials, Section F). Moreover, the model can be parametrized on the basis of available data. There are ample data for mean and variance of spVL and also most of the parameters can be confined to plausible ranges based on the literature.

Our study clearly supports that high heritability is compatible with the observed distribution of log spVL in HIV carriers. High heritability of spVL does not preclude that also the host genotype has a considerable effect on virus load. However, it does lead to the expectation that over the course of infection the capacity to induce higher spVL should increase considerably unless this capacity is only weakly linked to intrahost fitness. This sheds new light onto the mechanisms controlling viral load. There should be identifiable genetic variation in the virus population that is associated with viral load, and moreover, the loci associated with control of viral load should be weakly linked to intrahost fitness. Genome-wide association studies mapping viral genetic polymorphisms to variance in log spVL seem a natural approach to test this prediction. A recent study by Bartha et al. [38] was unable to identify any statistical associations, but was powered only to detect individual non-synonymous mutations with an effect size of >4% on heritability. Larger studies will thus be required to identify whether and which viral polymorphisms are associated with set point viral load.

## Methods

In the following sections we derive an analytical model that describes the change of mean and variance of spVL in the population of HIV carriers as a function of the heritability of spVL. We account for the virus and host effects by subdividing the phenotype (i.e. log spVL) into genetic and environmental/host components. Generally we denote the changes in mean and variance in the carrier, donor, and recipient populations with the subscripts *C*, *D* and *R*, respectively. We use greek letters for the parameters of the model and latin letters for the variables. When referring to the phenotype (i.e. log spVL) we use upper-case letters and when referring to the genotype we use lower-case letters.

### Distribution of log spVL in carrier population

The spVL in a patient is generally determined by viral genetic factors, host genetic factors, the environment and interactions between these factors. Since only the virus is transmitted from donors to recipients, we subsume all non-transmissible effects such as the host genetic factors, environmental effects and all interactions between host, virus and the environment generically under “environmental effects”, *e*. The transmissible effects due to the viral genotype are the “genotypic effects”, *g*. The “phenotype” spVL is then given by *g*+*e*.

We assume that the distribution of log spVL in the carrier population is given by a normal distribution $N(M_{C},V_{C})$, where *M*_*C*_ and *V*_*C*_ are the mean and variance, respectively. The transmission potential, defined as the overall probability of transmission of an HIV carrier integrated over the entire course of the disease, is assumed to be a function of log spVL which can be well approximated by a normal distribution $N(\mu_{o},\nu_{o})$ (see Fig. 2). Here *μ*_*o*_ is the log spVL at which the transmission potential is maximal and *ν*_*o*_ characterizes how strongly the transmission potential selects for transmission at *μ*_*o*_.

We assume that *g* and *e* are independent and normally distributed in the carrier population with $N(m_{C},v_{C})$ and $N(\mu_{e},\nu_{e})$, respectively. Here *m*_*C*_ and *v*_*C*_ are the variables that describe the mean and variance of the distribution of viral genotypes in the carrier population. Note that here the independence of *g* and *e* refers to the quantitative contribution of virus and host to spVL. Importantly, this independence does not imply an absence of virus genotype by host genotype interactions, such as an interaction between a particular viral epitope and a host HLA molecule. Genotype by genotype interactions are non-transmissible and thus subsumed in *e*. The parameters *μ*_*e*_ and *ν*_*e*_ describe mean and variance of the distribution of environmental effects, which comprise host effects, interactions and any non-transmissible effect. The distribution of phenotype log spVL in the carrier population is then given by a normal distribution with mean and variance,

$$M_{C}=m_{C}+\mu_{e},\;\text{and}\;V_{C}=v_{C}+\nu_{e}.$$ (4)

### Selection of donors

Selection for transmission acts on log spVL, i. e. on the sum of the genotypic and environmental effects, and is given by the transmission potential. Specifically, the probability of transmission for a given log spVL, *ϕ*, is given by (see Fig. 2B),

$$S\left(\phi \right)=\frac{1}{\sqrt{2\pi \nu_{o}}}e^{-\frac{{\left(\phi -\mu_{o}\right)}^{2}}{2\nu_{o}}}.$$ (5)

Applying the above transmission potential to the carrier population, we find that the genotype and phenotype in the donor population are again normally distributed (see Supplementary Materials, Equations B6 and B7). The donor genotype has mean and variance,

$$m_{D}=\frac{m_{C}\left(\nu_{e}+\nu_{o}\right)+\left(\mu_{o}-\mu_{e}\right)v_{C}}{v_{C}+\nu_{e}+\nu_{o}},\;\text{and}\;v_{D}=\frac{v_{C}\left(\nu_{e}+\nu_{o}\right)}{\nu_{e}+\nu_{o}+v_{C}}.$$ (6)

The donor phenotype has mean and variance (see Supplementary Materials, Equations B8 and B9),

$$M_{D}=\frac{M_{C}\nu_{o}+\mu_{o}V_{C}}{\nu_{o}+V_{C}},\;\text{and}\;V_{D}=\frac{V_{C}\nu_{o}}{\nu_{o}+V_{C}}.$$ (7)

Note, that the mean and variance of the environmental effects (i.e. the host effect) is not given by the differences between the phenotypic and genotypic values, because environment and genotype in the donors are correlated. This is because selection for transmission acts on the sum of environmental and genotypic effects. In other words selection for transmission selects a subset of viral genotypes and host genotypes, and host and viral genotypes are correlated, because selection operates on their combined effect.

### Transmission to recipients

When the virus is transmitted from the donor to the recipient population, the virus is “harvested” from a non-random distribution of environmental effects (and thus also from a non-random set of hosts). The harvested virus is then redistributed over a random set of new hosts/environments in the recipient population. Thus all environmental effects in the donor population are erased at transmission and the environmental contribution in the recipients is redrawn from $N(\mu_{e},\nu_{e})$. To account for the fact that the virus population experiences a strong bottleneck from recipient to donor, we assume that the viral genotype is not transmitted exactly from donor to recipient but instead is assumed to be randomly drawn out of a distribution of genotypes in the donor patient. Assuming that this distribution is normal with mean *m*_*D*_ and variance *ν*_*t*_ we obtain that both genotype and phenotype in the donor population are normally distributed. The recipient genotype has mean and variance,

$$m_{R}=m_{D},\;\text{and}\;v_{R}=v_{D}+\nu_{t}.$$ (8)

The recipient phenotype has mean and variance,

$$M_{R}=m_{R}+\mu_{e}^{0},\;\text{and}\;V_{R}=v_{R}+\nu_{e}^{0},$$ (9)

where $\mu_{e}^{0}$ and $\nu_{e}^{0}$ are the mean and variance of the host/environmental effects prior to infection. The environmental effects are redrawn randomly, because they are not inherited from one transmission to the next. Note, that we assume here that the bottleneck at transmission is neutral. This assumption does not imply that there is no selection at the transmission stage, but rather that the bottleneck is neutral with regard to the spVL that the transmitted strains will eventually cause. Any selection at and after transmission on the viral genotypic contribution to log spVL is subsumed in the next step, intrahost evolution.

### Intrahost evolution

After transmission the virus population in the recipient may change in a directed fashion according to intrahost evolution. Assuming that the overall change of the viral genotype due to intrahost evolution can be approximated by a normal distribution we find that the distribution of genotypes and phenotypes in the next generation of carriers, *C* ^′^ is again normal. The distribution of the genotypes has a mean and variance

$$m_{C^{'}}=m_{R}+\mu_{i},\;\text{and}\;v_{C^{'}}=v_{R}+\nu_{i}.$$ (10)

The parameter *μ*_*i*_ thus describes any genetic change in the virus that affects log spVL across all patients in the same way. The parameter *ν*_*i*_ describes genetic changes that affect log spVL in a manner that is specific to the patient, i.e. it describes the effect of changes of log spVL due to genetic interactions between virus and host. As the environmental effects comprise the immune response by the host, the mean and variance in environmental effects may change in coevolution with the virus through $\mu_{e}^{i}$ and $\nu_{e}^{i}$, respectively. Thus we obtain for mean and variance of the distribution of phenotypes,

$$M_{C^{'}}=m_{C^{'}}+\mu_{e}^{0}+\mu_{e}^{i},\;\text{and}\;V_{C^{'}}=v_{C^{'}}+\nu_{e}^{0}+\nu_{e}^{i}.$$ (11)

Note, that any selection for spVL at the transmission bottleneck can now be interpreted as a genotypic change that occurs during intrahost evolution. Thus the overall model is appropriate both for non-selective and selective bottlenecks.

### Heritability

Heritability, *h* ^2^, is defined as fraction of genotypic variance relative to phenotypic variance in the carrier population [39]. Thus we have,

$$h^{2}=\frac{v_{C}}{V_{C}}=1-\frac{\nu_{e}}{V_{C}}.$$ (12)

Heritability can be estimated in a parent-offspring regression [39], where *h* ^2^ is equal to the regression slope *b*. Donor-recipient pairs can be seen as parent-offspring pairs, where care must be taken since the donors are not randomly selected from the carrier population but are selected according to the transmission potential. Since, however, we are measuring the heritability of spVL and donors are selected based on spVL, the regression of recipients on selected donors is equal to heritability of spVL in carriers [24, 39].

### Mean and variance of log spVL at equilibrium

We now have a complete analytical description how mean and variance of log spVL change from the current to the next generation of carriers. The fact that the log spVL that maximizes the transmission potential and the mean of the distribution of log spVL in the carrier populations (see Fig. 2 and Fraser et al. [6]) are both around 4.5, we can assume that the process is roughly at equilibrium. In equilibrium we have that the mean and variance of the distribution of phenotypes does not change, i.e. *M*_*C*^′^_ = *M*_*C*_ and *V*_*C*^′^_ = *V*_*C*_. This will be fulfilled if the genetic and environmental contributions are also at equilibrium, implying in particular that $\mu_{e}=\mu_{e}^{0}+\mu_{e}^{i}$ and $\nu_{e}=\nu_{e}^{0}+\nu_{e}^{i}$ (see Supplementary Materials section C.1). Using Equation 12 we can express the equilibrium mean and variance of log spVL as a function of *ν*_*e*_, the variance of the contribution of the host/environment to log spVL (see Supplementary Materials, Equations C9 and C10),

$${\tilde{M}}_{C}\;=\;\mu_{o}+\mu_{i}\left(1+\frac{\nu_{e}+\nu_{o}}{{\tilde{V}}_{C}-\nu_{e}}\right),$$ (13)

$${\tilde{V}}_{C}\;=\;\frac{\nu_{t}+\nu_{i}}{2}\left(1+\sqrt{1+4\frac{\nu_{e}+\nu_{o}}{\nu_{t}+\nu_{i}}}\right)+\nu_{e}.$$ (14)

or as a function the heritability *h* ^2^ (see Supplementary Materials, Equations C12 and C13),

$${\tilde{M}}_{C}\;=\;\mu_{o}+\mu_{i}\left(1+\frac{(1-h^{2}){\tilde{V}}_{C}+\nu_{o}}{h^{2}{\tilde{V}}_{C}}\right),$$ (15)

$${\tilde{V}}_{C}\;=\;\frac{\nu_{t}+\nu_{i}}{2{\left(h^{2}\right)}^{2}}\left(1+\sqrt{1+\frac{4{\left(h^{2}\right)}^{2}\nu_{o}}{\nu_{t}+\nu_{i}}}\right).$$ (16)

## Supporting Information

S1 Text Supplementary Methods and Figures. (PDF)
