# Supplementary material for: High Heritability Is Compatible with the Broad Distribution of Set Point Viral Load in HIV Carriers
Source: PLoS Pathog. 2015 Feb 6;11(2):e1004634. doi: 10.1371/journal.ppat.1004634 (PMC4450065; doi:10.1371/journal.ppat.1004634)
Supplement: S1 Text — (PDF) [file ppat.1004634.s001.pdf]

High heritability is compatible with the broad distribution of set point viral load in HIV carriers: Supplementary Text S1

Sebastian Bonhoeffer[1,*], Christophe Fraser[2], and Gabriel E. Leventhal[1]

[1]Institute of Integrative Biology, ETH Zurich, Switzerland
[2]Department of Infectious Disease Epidemiology, Imperial College London, London, United Kingdom
[*]corresponding author. E-mail: sebastian.bonhoeffer@env.ethz.ch

Contents

A Distribution of genotype and phenotype in the different populations along a full replication cycle **2**
- A.1 Carrier population . . . 2
- A.2 Donor population . . . 2
- A.3 Recipient population . . . 3
- A.4 Evolved population (after intrahost evolution) . . . 4

B Analytical solution assuming normal distributions **4**
- B.1 Carriers . . . 4
- B.2 Selected donors . . . 5
- B.3 Recipients . . . 6
- B.4 New carriers . . . 6

C Equilibrium solutions for mean and variance of spVL **7**
- C.1 Equilibrium of environmental factors . . . 8

D Connection to integral projection models **9**

E Viral load in Geskus et al. (1) **9**

F Deviations from normality **10**
- F.1 Exact transmission potential . . . 10
- F.2 Skewness in intrahost evolution and transmission bottleneck . . . 11
- F.3 Influence of the acute and AIDS phase on the transmission potential . . . 12

A Distribution of genotype and phenotype in the different populations along a full replication cycle

In this section we will derive expressions for the distributions of genotypes g, environment e and phenotype ϕ in the populations of carriers C, donors D, recipients R and new carriers E. The phenotype ϕ refers here to the log set point virus load (log spVL). The genotype g refers to the virus and the environment e refers to all non-transmissible contribution to log spVL, i.e. the contributions from the host genotype, from the interactions between host and viral genotypes and from the environment. Generally, $p_{x,Y}$ will denote the distribution of $x \in \{g, e, \phi\}$ in the population $Y \in \{C, D, R, E\}$. The phenotype $\phi(g, e)$ is a function of the genotype g and the environment e. The simplest assumption is that g and e contribute additively,

$$\phi(g, e) = g + e. \tag{A1}$$

A.1 Carrier population

Let the joint distribution of genotypes and environments in the carrier population be $p_{ge,C}(g, e)$. Assuming that genotypes and environments are independently distributed we have,

$$p_{ge,C}(g, e) = p_{g,C}(g)p_{e,C}(e). \tag{A2}$$

The distribution of the phenotype ϕ in the carrier population is,

$$\begin{aligned} p_{\phi,C}(\phi) &= \iint p_{ge,C}(g, e|\phi)p_{g,C}(g)p_{e,C}(e)\, dgde && \text{(A3)} \\ &= \iint \delta(\phi - (g + e))p_{g,C}(g)p_{e,C}(e)\, dgde && \text{(A4)} \\ &= \int p_{g,C}(g)p_{e,C}(\phi - g)\, dg && \text{(A5)} \\ &= [p_{g,C} * p_{e,C}](\phi). && \text{(A6)} \end{aligned}$$

Here, δ is the Dirac-delta function and the asterisk denotes the convolution of the distributions $p_{g,C}$ and $p_{e,C}$.

A.2 Donor population

Donors are selected from the current distribution of carriers according to their fitness $S(\phi)$ which depends on their phenotype $\phi = g + e$. The joint distribution of g and e in selected donors is,

$$p_{ge,D}(g, e) = \frac{1}{Z_s} p_{ge,C}(g, e)S(g + e) = \frac{1}{Z_s} p_{g,C}(g)p_{e,C}(e)S(g + e), \tag{A7}$$

where Z_s is a normalization constant,

$$\begin{aligned} Z_s &= \iint p_{ge,C}(g, e)S(g + e)dgde = \iint p_{g,C}(g)p_{e,C}(e)S(g + e)dedg && \text{(A8)} \\ &= \iint p_{g,C}(g)p_{e,C}(\phi - g)S(\phi)d\phi dg && \text{(A9)} \\ &= \int p_{\phi,C}(\phi)S(\phi)d\phi. && \text{(A10)} \end{aligned}$$

We can then write the joint distribution of genotypes g and phenotypes ϕ in the selected donors,

$$p_{g\phi,D}(g,\phi) = \int p_{ge,D}(g,e)\delta(\phi-(g+e))de \tag{A11}$$

$$= \frac{1}{Z_s}\int p_{g,C}(g)p_{e,C}(e)S(g+e)\delta(\phi-(g+e))de \tag{A12}$$

$$= \frac{1}{Z_s}p_{g,C}(g)p_{e,C}(\phi-g)S(\phi). \tag{A13}$$

The distribution of genotypes irrespective of the phenotype then is $p_{g,D}(g,\phi)$ marginalized over ϕ,

$$p_{g,D}(g) = \int p_{g,D}(g,\phi)d\phi = \frac{1}{Z_s}\int p_{g,C}(g)p_{e,C}(\phi-g)S(\phi)d\phi. \tag{A14}$$

Similarly, the distribution of the phenotype ϕ in the selected donors is,

$$p_{\phi,D}(\phi) = \int p_{g,D}(g,\phi)dg = \frac{1}{Z_s}\int p_{g,C}(g)p_{e,C}(\phi-g)S(\phi)dg = \frac{1}{Z_s}[p_{g,C} * p_{e,C}](\phi)S(\phi). \tag{A15}$$

A.3 Recipient population

The distribution of genotypes in the recipient population is shaped by the transmission function $\mathcal{T}(g_R, g_D)$, which determines the genotype g_R of a recipient given that the genotype of the donor was g_D. So the distribution of g in the recipients is $\mathcal{T}$ integrated over all genotypes in the donor population,

$$p_{g,R}(g_R) = \int \mathcal{T}(g_R,g_D)p_{g,D}(g_D)\,dg_D \tag{A16}$$

$$= \frac{1}{Z_s}\iint \mathcal{T}(g_R,g_D)p_{g,C}(g_D)p_{e,C}(\phi-g_D)S(\phi)\,d\phi\,dg_D. \tag{A17}$$

We can write the distribution of phenotype in the recipient population as,

$$p_{\phi,R}(\phi_R) = \int p_{g,R}(g_R)p_{e,R}(\phi_R-g_R)dg_R \tag{A18}$$

$$= \iint \mathcal{T}(g_R,g_D)p_{g,D}(g_D)p_{e,R}(\phi_R-g_R)dg_D dg_R \tag{A19}$$

$$= \int p_{g,D}(g_D)dg_D \int \mathcal{T}(g_R,g_D)p_{e,R}(\phi_R-g_R)dg_R \tag{A20}$$

$$= \int p_{g,D}(g_D)[\mathcal{T} * p_{e,R}](\phi_R,g_D)dg_D. \tag{A21}$$

Inserting equation (A14),

$$p_{\phi,R}(\phi_R) = \frac{1}{Z_s}\iint [\mathcal{T} * p_{e,R}](\phi,g)\,p_{g,C}(g)p_{e,C}(\phi'-g)S(\phi')\,d\phi'\,dg. \tag{A22}$$

A.4 Evolved population (after intrahost evolution)

Let $\mathcal{E}_g(g_E, g_R)$ be the function that evolves the genotype within the host. The distribution of genotypes in the evolved recipients is then,

$$p_{g,E}(g_E) = \int \mathcal{E}_g(g_E, g_R) p_{g,R}(g_R) dg_R. \tag{A23}$$

Inserting equation (A17),

$$p_{g,E}(g_E) = \frac{1}{Z_s} \iiint \mathcal{E}_g(g_E, g_R) \mathcal{T}(g_R, g_D) p_{g,C}(g_D) p_{e,C}(\phi - g_D) S(\phi)\, d\phi\, dg_D\, dg_R. \tag{A24}$$

Due to the evolution of the virus genetics, the host-virus interactions can change. This would result in a change in the distribution of e in the evolved population. Let $\mathcal{E}_e(e_E, e_R)$ be the function that evolves the interactions within the host. The distribution of environmental factors in the evolved recipients is then,

$$p_{e,E}(e_E) = \int \mathcal{E}_e(e_E, e_R) p_{e,R}(e_R) de_R. \tag{A25}$$

We can write the distributions of phenotypes as,

$$\begin{aligned}
p_{\phi,E}(\phi_E) =& \int p_{g,E}(g_E) p_{e,E}(\phi_E - g_E)\, dg_E && \text{(A26)} \\
=& \frac{1}{Z_s} \int \cdots \int \mathcal{E}_g(g_E, g_R) \mathcal{T}(g_R, g_D) p_{g,C}(g_D) p_{e,C}(\phi - g_D) S(\phi) \\
& \times\ \mathcal{E}_e(\phi_E - g_E, e_R) p_{e,R}(e_R)\, de_R\, d\phi\, dg_D\, dg_R\, dg_E && \text{(A27)} \\
=& \frac{1}{Z_s} \iiint \mathcal{T}(g_R, g_D) p_{g,C}(g_D) p_{e,C}(\phi - g_D) S(\phi)\, d\phi\, dg_D\, dg_R \\
& \times \int \left[\mathcal{E}_g * \mathcal{E}_e\right](\phi_E; g_R, e_R) p_{e,R}(e_R)\, de_R. && \text{(A28)}
\end{aligned}$$

B Analytical solution assuming normal distributions

While the above expressions hold for any distribution, the integral cannot be solved in the general case. If we assume normal distributions for all the different processes, we are able to derive closed-form expressions.

B.1 Carriers

We assume that the distributions $p_{g,C}$ and $p_{e,C}$ are normally distributed,

$$p_{g,C} = \frac{1}{\sqrt{2\pi v_C}} \exp\left\{-\frac{(m_C - g)^2}{2v_C}\right\}, \tag{B1}$$

$$p_{e,C} = \frac{1}{\sqrt{2\pi \nu_e}} \exp\left\{-\frac{(\mu_e - e)^2}{2\nu_e}\right\}. \tag{B2}$$

Here, (m_C, v_C) and (μ_e, ν_e) are the means and variances of the genotype and environmental distributions respectively.

Since the convolution of two Gaussian distributions with means μ_1 and μ_2 and variances σ_1^2 and σ_2^2 is also a Gaussian with mean $\mu_{12} = \mu_1 + \mu_2$ and variance $\sigma_{12}^2 = \sigma_1^2 + \sigma_2^2$, the distribution of phenotypes in the carrier population $p_{\phi,C}$ is also normal with mean,

$$M_C = m_C + \mu_e, \tag{B3}$$

and variance,

$$V_C = v_C + \nu_e. \tag{B4}$$

B.2 Selected donors

Additionally, the product of two Gaussians is also a Gaussian (not necessarily normalized) with mean,

$$\mu_p = \frac{\mu_1\sigma_2^2 + \mu_2\sigma_1^2}{\sigma_1^2 + \sigma_2^2},$$

and variance,

$$\sigma_p^2 = \frac{\sigma_1^2\sigma_2^2}{\sigma_1^2 + \sigma_2^2}.$$

Thus $p_e \equiv p_{e,C}$ is symmetric around the mean μ_e such that,

$$p_e(\phi - g) = p_e((g + 2\mu_e) - \phi),$$

and equation (A14) becomes,

$$p_{g,D}(g) = \frac{1}{Z_s} p_{g,C}(g)[p_e * S](g + 2\mu_e). \tag{B5}$$

The convolution of p_e and S has mean $\mu_e + \mu_o$ and variance $\nu_e + \nu_o$. If we write,

$$A(g) = [p_e * S](g + 2\mu_e),$$

then A is a Gaussian with variance $\nu_e + \nu_o$ and mean $\mu_e + \mu_o - 2\mu_e = \mu_o - \mu_e$. From the product formula above, $p_{g,D} \sim \mathcal{N}(m_D, v_D)$,

$$m_D = \frac{m_C(\nu_e + \nu_o) + (\mu_o - \mu_e)v_C}{v_C + \nu_e + \nu_o}, \tag{B6}$$

$$v_D = \frac{v_C(\nu_e + \nu_o)}{v_C + \nu_e + \nu_o}. \tag{B7}$$

The distribution of phenotypes in the donor population follows from equation (A15) directly. So, $p_{\phi,D} \sim \mathcal{N}(M_D, V_D)$,

$$M_D = \frac{M_C\nu_o + \mu_o V_C}{\nu_o + V_C}, \tag{B8}$$

$$V_D = \frac{V_C\nu_o}{V_C + \nu_o}. \tag{B9}$$

B.3 Recipients

The transmission function $\mathcal{T}$ determines the viral genotype of the recipient, given that the genotype of the donor was g_R. We assume that $\mathcal{T}$ is normally distributed around g_R with variance ν_t. Thus equation (A17) becomes

$$p_{g,R}(g_R) = \int p_t(g_R - g_D)p_{g,D}(g_D)\, dg_D,$$

where p_t is a Gaussian with zero mean and variance ν_t. This integral is again a convolution, such that $p_{g,R} \sim \mathcal{N}(m_R, v_R)$ with,

$$m_R = m_D, \tag{B10}$$

$$v_R = v_D + v_t. \tag{B11}$$

Equivalently for the phenotype distribution in the recipients, from equation (A21),

$$p_{\phi,R}(\phi_R) = \int p_{t+e}(\phi_R - g_D)p_{g,D}(g_D)\, dg_D,$$

where p_{t+e} is a Gaussian with mean μ_e^0 and variance $v_t + \nu_e^0$. Thus the convolution is again Gaussian and $p_{\phi,R} \sim \mathcal{N}(M_R, V_R)$,

$$M_R = m_D + \mu_e^0, \tag{B12}$$

$$V_R = v_D + v_t + \nu_e^0. \tag{B13}$$

B.4 New carriers

The same as for transmission, we assume that the evolver functions for the viral and environmental contribution is $\mathcal{E}_g \sim \mathcal{N}(g_R + \mu_i, \nu_i^g)$ and $\mathcal{E}_e \sim \mathcal{N}(e_R + \mu_e^i, \nu_e^i)$, respectively. The evolved population of new carriers has a genotype distribution given by equation (A23).

$$p_{g,E}(g_E) = \int p_{Eg}((g_E - \mu_i) - g_R)p_{g,R}(g_R)dg_R,$$

where p_E has mean zero and variance ν_i^g, such that $p_{g,E} \sim \mathcal{N}(m_{C'}, v_{C'})$,

$$m_{C'} = m_R + \mu_i, \tag{B14}$$

$$v_{C'} = v_R + \nu_i^g. \tag{B15}$$

The distribution of phenotypes in the evolved population as a function of the distribution in the recipient population is,

$$p_{\phi,E}(\phi_E) = \iiint p_{g,R}(g_R)\mathcal{E}_g(g_E, g_R)p_{e,R}(e_R)\mathcal{E}_e(\phi_E - g_E, e_R)\, dg_R\, dg_E\, de_R.$$

Let $p_{Ee}(x)$ be a normal distribution with mean zero and variance ν_e^i,

$$p_{\phi,E}(\phi_E) = \iint p_{g,R}(g_R)\mathcal{E}_g(g_E, g_R) \int p_{e,R}(e_R)p_{Ee}((\phi_E - g_E - \mu_e^i) - e_R)\, dg_R\, dg_E\, de_R \tag{B16}$$

$$= \iint p_{g,R}(g_R)\mathcal{E}_g(g_E, g_R)f_1(\phi_E - g_E)\, dg_R\, dg_E, \tag{B17}$$

with f_1 a normal distribution with mean $\mu_e^0 + \mu_e^i$ and variance $\nu_e^0 + \nu_e^i$. Integrating the convolutions further,

$$p_{\phi,E}(\phi_E) = \int f_1(\phi_E - g_E)\, dg_E \int p_{g,R}(g_R) p_{Eg}(g_E - g_R - \mu_i)\, dg_R \tag{B18}$$

$$= \int f_1(\phi_E - g_E) f_2(g_E)\, dg_E, \tag{B19}$$

where f_2 is a normal distribution with mean $m_R + \mu_i$ and variance $v_R + \nu_i^g$.
The distribution of the phenotype follows from the convolution of f_1 and f_2, such that $p_{\phi,E} \sim \mathcal{N}(M_{C'}, V_{C'})$,

$$M_{C'} = m_R + \mu_i + \mu_e^0 + \mu_e^i, \tag{B20}$$

$$V_{C'} = v_R + \nu_i^g + \nu_e^0 + \nu_e^i \tag{B21}$$

C Equilibrium solutions for mean and variance of spVL

Concerning log spVL under the assumption of normal distributions, we have the following expressions for the distribution of log spVL in the current carriers and the carriers in the following generation,

$$\phi_C \sim \mathcal{N}\left(m_C + \mu_e, v_C + \nu_e\right), \tag{C1}$$

$$\phi_{C'} \sim \mathcal{N}\left(\frac{m_C(\nu_e + \nu_o) + (\mu_o - \mu_e)v_C}{v_C + \nu_e + \nu_o} + \mu_i + \mu_e^0 + \mu_e^i, \frac{v_C(\nu_e + \nu_o)}{v_C + \nu_e + \nu_o} + \nu_t + \nu_i^g + \nu_e^0 + \nu_e^i\right). \tag{C2}$$

The system is said to be in equilibrium when the distribution in phenotype not longer changes from one generation to the next, thus,

$$m_C + \mu_e = \frac{m_C(\nu_e + \nu_o) + (\mu_o - \mu_e)v_C}{v_C + \nu_e + \nu_o} + \mu_i + \mu_e^0 + \mu_e^i, \tag{C3}$$

$$v_C + \nu_e = \frac{v_C(\nu_e + \nu_o)}{v_C + \nu_e + \nu_o} + \nu_t + \nu_i^g + \nu_e^0 + \nu_e^i. \tag{C4}$$

From equation (C4) we readily find the equilibrium solution for v_C,

$$\tilde{v}_C = \frac{\nu_t + \nu_i + (\nu_e^0 + \nu_e^i - \nu_e)}{2}\left(1 \pm \sqrt{1 + 4\frac{\nu_e + \nu_o}{\nu_t + \nu_i + (\nu_e^0 + \nu_e^i - \nu_e)}}\right). \tag{C5}$$

The equilibrium solution of m_C as a function of v_C is then,

$$\tilde{m}_C = (\mu_o - \mu_e) + \left(\mu_i + \mu_e^0 + \mu_e^i - \mu_e\right)\left(1 + \frac{\nu_e + \nu_o}{\tilde{v}_C}\right). \tag{C6}$$

If we assume that at equilibrium, the distributions of environmental factors no longer change from one generation of carriers to the next, then,

$$\mu_e' \equiv \mu_e^0 + \mu_e^i = \mu_e, \tag{C7}$$

$$\nu_e' \equiv \nu_e^0 + \nu_e^i = \nu_e, \tag{C8}$$

where the prime signifies the values of mean and variance of environmental factors in the new generation of carriers. Thus the equilibrium solutions for the phenotype distribution are,

$$\tilde{M}_C = \tilde{m}_C + \mu_e = \mu_o + \mu_i \left(1 + \frac{\nu_e + \nu_o}{\tilde{V}_C - \nu_e}\right), \tag{C9}$$

$$\tilde{V}_C = \tilde{v}_C + \nu_e = \frac{\nu_t + \nu_i^g}{2}\left(1 \pm \sqrt{1 + 4\frac{\nu_e + \nu_o}{\nu_t + \nu_i^g}}\right) + \nu_e. \tag{C10}$$

We can express the equilibrium solutions in terms of the heritability h^2, where

$$\nu_e = (1 - h^2)\tilde{V}_C. \tag{C11}$$

Inserting into equation (C10),

$$\tilde{V}_C = \frac{\nu_t + \nu_i}{2}\left(1 + \sqrt{1 + 4\frac{(1-h^2)\tilde{V}_C + \nu_o}{\nu_t + \nu_i}}\right) + (1 - h^2)\tilde{V}_C.$$

By rearranging the terms we get,

$$\tilde{V}_C h^2 \frac{2}{\nu_t + \nu_i} - 1 = \sqrt{1 + 4\frac{(1-h^2)\tilde{V}_C + \nu_o}{\nu_t + \nu_i}}.$$

Squaring both sides yield the quadratic equation,

$$\tilde{V}_C^2 - \frac{\nu_t + \nu_i}{(h^2)^2}\tilde{V}_C - \frac{(\nu_t + \nu_i)\nu_o}{(h^2)^2} = 0.$$

that has the solutions,

$$\tilde{V}_C = \frac{\nu_t + \nu_i}{2(h^2)^2}\left(1 \pm \sqrt{1 + \frac{4(h^2)^2\nu_o}{\nu_t + \nu_i}}\right).$$

Keeping only the non-negative solution and inserting equation (C11) in the expression for $\tilde{M}_C$,

$$\tilde{M}_C = \mu_o + \mu_i\left(1 + \frac{(1-h^2)\tilde{V}_C + \nu_o}{\tilde{V}_C - (1-h^2)\tilde{V}_C}\right) = \mu_o + \mu_i\left(1 + \frac{(1-h^2)\tilde{V}_C + \nu_o}{h^2\tilde{V}_C}\right), \tag{C12}$$

$$\tilde{V}_C = \frac{\nu_t + \nu_i}{2(h^2)^2}\left(1 + \sqrt{1 + \frac{4(h^2)^2\nu_o}{\nu_t + \nu_i}}\right). \tag{C13}$$

C.1 Equilibrium of environmental factors

In the main text we argue that there is good evidence that the phenotypic distribution of spVL is approximately in equilibrium, and thus $M_{C'} = M_C$ and $V_{C'} = V_C$. In the above derivation, we assume that this also implies an equilibrium of the environmental factors,

$$\mu_e' \equiv \mu_e^0 + \mu_e^i = \mu_e,$$
$$\nu_e' \equiv \nu_e^0 + \nu_e^i = \nu_e.$$

It is straightforward to see that if both the distributions for g and e are in equilibrium, then the distribution for ϕ is also in equilibrium. There are, however, certain special cases that can be considered where an equilibrium of ϕ does not imply an equilibrium of g and e. Firstly, the distribution of ϕ might converge faster to an equilibrium value than the distributions of g and e. This would imply that the contributions of the virus and the environment to the variance in spVL might still be changing over time. Consequently, heritability may also still be changing over time. Secondly, the contributions of g and e may be diverging in opposite directions, such that the change in the distribution of g cancels out the change in the distribution of e on the population level. This scenario, however, is unlikely as it requires the viral and host/environmental factors that influence spVL to increase or decrease indefinitely. Thirdly, the change in g and e on the population level is described by a stable limit cycle, such that the distribution in spVL in the population is constant through time, $\phi(t) = g(t) + e(t) \equiv \tilde{\phi}$. While stable limit cycles can appear in theoretical models, they are rarely observed in real complex biological systems, due to the delicate balance required between the variables. Furthermore, this balance has to be maintained on a population level, which would require some sort of synchrony between the evolutionary changes happening in each individual host. We therefore argue that it is most conceivable that the equilibrium of spVL in the population also implies an equilibrium of the distribution of viral and environmental effects.

D Connection to integral projection models

Our description of the distributions of log spVL change over generations has strong parallels to *integral projection models* used in ecology to describe how the composition of population with continuous traits changes over discrete time (2–4). In this formalism, the number of individuals with trait y in generation $t+1$ is given by (2),

$$n(y, t+1) = \int_{\Omega} k(y, x) n(x, t) dx. \tag{D1}$$

Here, $k(y, x)$ is called the kernel and defines the number of offspring with trait y produced by an offspring of trait x in generation t.

Heritability can be viewed as the regression of offspring on parents, i.e. new carriers on old carriers. As we assume the distribution of log spVL in carriers to be normal, the conditional distribution of log spVL in new carriers given an log spVL current carriers is,

$$p(\phi_{C'} | \phi_C = \varphi) \sim \mathcal{N}\left(M_C + \sqrt{\frac{V_C}{V_{C'}}}\rho(\varphi - M_{C'}), (1-\rho^2)V_C\right), \tag{D2}$$

where $\rho = \sqrt{V_{C'}/V_C} h^2$ is the correlation coefficient between carriers in subsequent generations. Thus the projection kernel $k(\phi_{C'}, \phi_C) = p(\phi_{C'} | \phi_C)$.

E Viral load in Geskus et al. (1)

We extracted the viral load measurements from the pdf file of Geskus et al. (1) to provide a further estimate of mean and variance of viral load. This study is also based on the Amsterdam cohort, but the patient population is not identical to the one used in Fraser et al. (5). Excluding

measurements that were under the detection limit we estimate a mean of 4.22 logs with a variance of 0.59. The fitted line in figure S1 shows that the distribution is well approximated by a normal distribution, although a statistical test reveals a significant deviation from normality. Note that the viral loads reported in Geskus et al. (1) are not spVLs, but include also repeated measurements from individual patients. As a consequence the sample variance is likely an overestimate of the real variance of spVL.

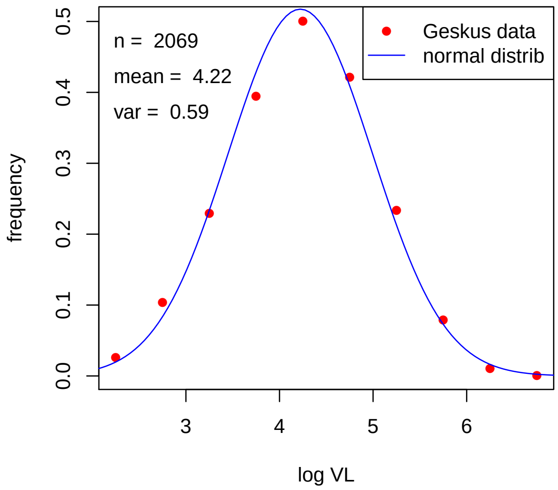

Figure S1. Distribution of spVL in donors and recipients in Geskus et al. (1). The plot is confined to viral load measured between years 1 and 5 after serovonversion.

F Deviations from normality

F.1 Exact transmission potential

In this section we assess to what degree the normal approximation to the transmission potential (TP) results in a distribution of spVL in HIV carriers that is different from using the TP as reported in Fraser et al. (5). We also account for uncertainty in the transmission potential by accounting for the confidence intervals in the reported TP. To this end we simulate 20 reproduction cycles (i.e. selection for donors, transmission and intrahost evolution) in a population of $N = 10^5$ individuals. At each reproduction cycle the number the donors of the N recipients are selected in the following manner:

(a) The maximum likelihood estimate for the number of infections caused by an individual with spVL v, as well as the upper and lower bounds of the confidence are determined by linear interpolation of the TP from (5).

(b) We then construct a triangular distribution for the probability of x secondary infections at spVL v between the lower $x_{\rm lo}$ and upper $x_{\rm hi}$ bounds of the confidence interval, such that

the probability of x secondary infections fulfils $p(x_{\mathrm{lo}}|v) = p(x_{\mathrm{hi}}|v) = 0$ and $\mathrm{argmax}_x p(x|v) = x_{\mathrm{ML}} = \mathrm{TP}(v)$. The value of $p(x_{\mathrm{ML}})$ is such that $\int_{x_{\mathrm{lo}}}^{x_{\mathrm{hi}}} p(x)dx = 1$.

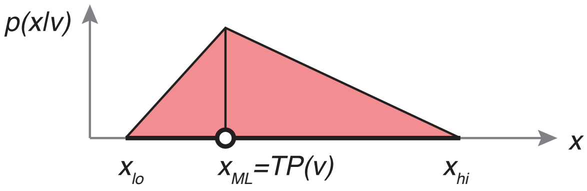

(c) The number of secondary infections x_i at the current reproduction cycle for each individual i is then sampled from the constructed distribution for each corresponding spVL v_i.

(d) Donors for all new recipients are picked randomly from the donor population with probability proportional to x_i.

The simulated distribution of spVL in carriers after 20 cycles is shown in Figure S2. The normal approximation is in very good agreement with the simulated distribution.

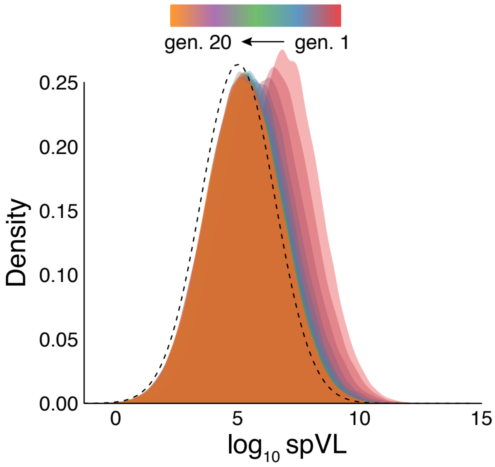

Figure S2. Simulated distribution of spVL in HIV carriers after 20 reproduction cycles when using the exact transmission potential together with the reported confidence intervals. Other parameters are $\mu_o = 4.5$, $\nu_o = 1$, $\mu_e = 3$, $\nu_e = 1$, $\mu_i = 0.2$, $\nu_i = 0.3$, $\nu_t = 0.2$. The starting population is assumed normally distributed with mean $m_g = 4$ and $v_g = 0.4$. The dashed line shows the equilibrium under the normal approximation to the transmission potential.

F.2 Skewness in intrahost evolution and transmission bottleneck

To test the effect of deviations from normality of the processes of intrahost evolution and the transmission bottleneck we sampled from a skew-normal instead of a normal distribution for

both processes. The skew-normal distribution is characterized by a location, a shape and a scale parameter that together define mean, variance and skewness of the distribution. If the shape parameter is zero, the distribution has no skewness and reduces to normal distribution. To sample from the skew-normal distribution we used the `rsnorm` function of the VGAM package in R (6). Figure S3 shows the effect of skewness in processes of intrahost evolution and the transmission bottleneck mean, variance and skewness of the spVL distribution in the carrier population by varying skewness in both processes from -0.9 to 0.9. The key result is that the analytical results for mean and variance of the spvL distribution remain excellent approximations even for strong skewness in the processes of intrahost evolution and the transmission bottleneck.

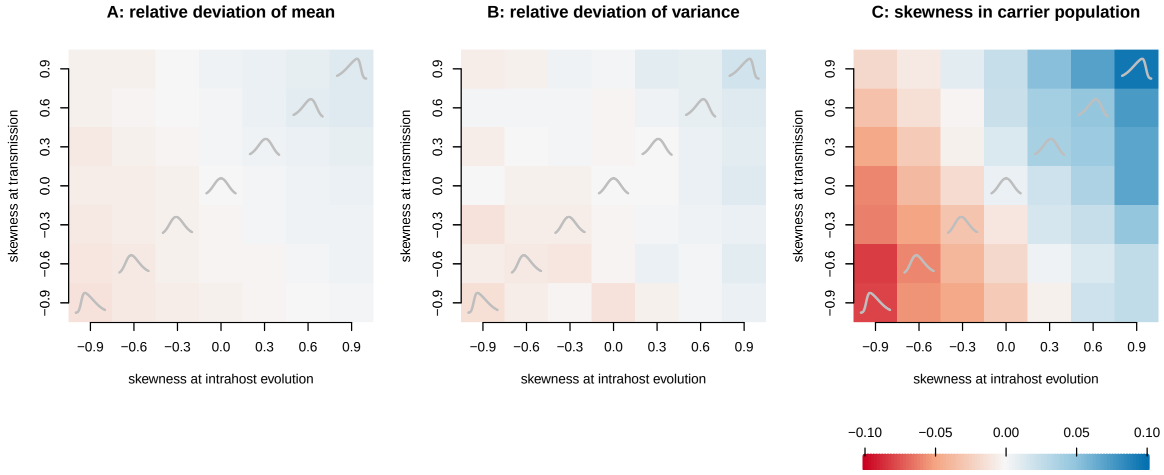

Figure S3. The effect of skewness in the processes of intrahost evolution and the transmission bottleneck on mean, variance and skewness of simulated distributions of spVL in carriers. Panel A shows the relative deviation of the computed mean from the analytical mean (eq. C12), i.e. the difference of computed and analytical mean divided by the analytical mean. Panel B shows the corresponding relative deviation from the analytical variance (eq. C13). Panel C shows the skewness of the distribution of spVL in the carrier population. The grey lines show distributions with the corresponding level of skewness. The color legend applies to all panels. Generally the relative deviation of mean and variance remains below a few percent even for large skewness in the processes of intrahost evolution and the transmission bottleneck. Also the absolute level of skewness in the simulated distributions (panel C) remains below 0.1. Taken together this indicates that even strongly skew processes lead to small effects on the resulting distribution of spVL in HIV carriers. Parameters of the simulation are $\mu_o = 4.5, \nu_o = 1, \mu_e = 3, \nu_e = 1, \mu_i = 0.2, \nu_i = 0.3, \nu_t = 0.2$. The population size used in the simulation is 200000.

F.3 Influence of the acute and AIDS phase on the transmission potential

One concern regarding the transmission potential from Fraser et al. (5) is that it neglects transmission from the acute and the AIDS phase of the infection. This is addressed in more detail in the supplementary material of Fraser et al. (5). As described therein the required correction de-

pends on the assumed model of sexual mixing and partner exchange rate. One way to account for the contribution of these phases is to add a constant term to the transmission potential. This term was estimated in Fraser et al. (5) to be 0.67 (0.32-1.23 95% c. i.) for primary infection and 0.50 (0.31-0.96 95% c. i.) for pre-AIDS/AIDS. A reasonable range for this constant, c, is thus $[0, 2]$.

We performed simulations to compare the equilibrium mean and variance for a transmission potential with a constant c to the analytical expression obtained assuming $c = 0$ (see figure S4). The simulations show that both mean and variance increase with increasing c. Adding a constant to the transmission potential results in overall weaker selection for viral load. This leads to a general increase in variance. The mean increases because the transmission potential is weaker in opposing the force of intrahost evolution towards higher spVL.

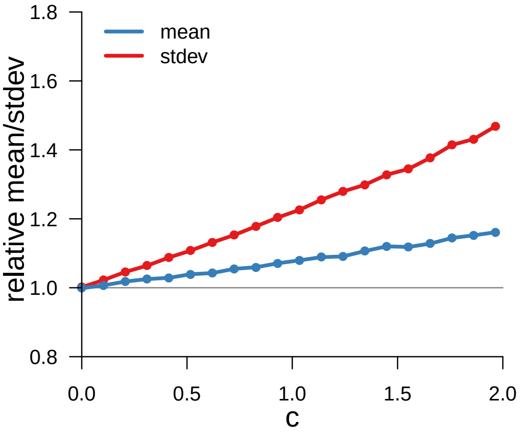

Figure S4. The effect of adding a contribution of the acute and AIDS phases to the overall transmission potential. We show the relative increase of mean and standard deviation compared to the analytical solution (eqs. C12 and C13) as a function of the constant c that is added to the transmission potential. This constant c spans a realistic range of contributions from the acute and AIDS phase as described in Fraser et al. (5). Parameters of the simulation are $\mu_o = 4.5, \nu_o = 1, \mu_e = 3, \nu_e = 1, \mu_i = 0.2, \nu_i = 0.3, \nu_t = 0.2$.

Furthermore, we tested the effect a corrected transmission potential by repeating the rejection sampling procedure using $c = 1.2$ (see figure S5). Using a corrected transmission potential generally narrows down the acceptable parameter ranges (because of the effect of increasing variance and mean shown in figure S4). The areas of highest posterior probability remain in regions of high heritability. Thus, in summary, modifying the transmission potential to account for the contributions of the acute and AIDS phase does not change the two key conclusions, namely that high heritability is the most parsimonious explanation for the observed mean and variance of spVL and that the forces of intrahost evolution must be weak.

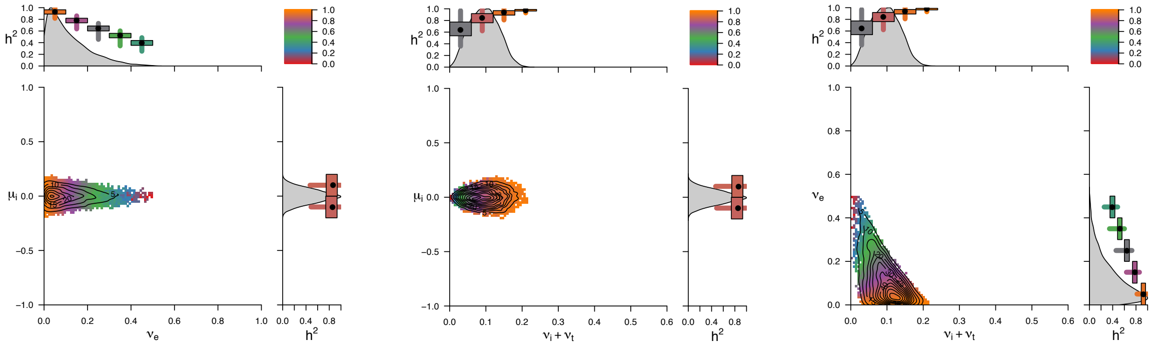

Figure S5. Posterior distribution of parameters from the rejection sampler assuming a transmission potential plus a constant $c = 1.2$. The figure is analogous to figure 3 in the main text. Since no analytical solutions are available for the modified transmission potential we performed simulations to measure the approximate equilibrium mean and variance. Because of the higher computational demands we sampled $40'000$ random sets of parameter values from these restricted priors: $0 < \nu_e < 0.6; 0 < \mu_i < 0.3; 0 < \nu_i, \nu_t < 0.15$. For comparison, however, we plot the accepted parameters over the same range as in figure 3 in the main text.

References

1. Geskus RB, Prins M, Hubert JB, Miedema F, Berkhout B, et al. (2007) The HIV RNA setpoint theory revisited. Retrovirology 4: 65. doi:10.1186/1742-4690-4-65.

2. Easterling MR, Ellner SP, Dixon PM (2000) Size-specific sensitivity: Applying a new structured population model. Ecology 81: 694–708.

3. Ellner SP, Rees M (2006) Integral Projection Models for Species with Complex Demography. Am Nat 167: 410–428.

4. Coulson T, MacNulty DR, Stahler DR, vonHoldt B, Wayne RK, et al. (2011) Modeling effects of environmental change on wolf population dynamics, trait evolution, and life history. Science 334: 1275–8. doi:10.1126/science.1209441.

5. Fraser C, Hollingsworth TD, Chapman R, de Wolf F, Hanage WP (2007) Variation in HIV-1 set-point viral load: epidemiological analysis and an evolutionary hypothesis. Proc Natl Acad Sci U S A 104: 17441–6. doi:10.1073/pnas.0708559104.

6. Yee TW (2013) VGAM: Vector Generalized Linear and Additive Models. R package version 0.9-2.
